# A Collaborative and Ubiquitous System for Fabricating Dental Parts Using 3D Printing Technologies

**DOI:** 10.3390/healthcare7030103

**Published:** 2019-09-06

**Authors:** Yu-Cheng Wang, Toly Chen, Yu-Cheng Lin

**Affiliations:** 1 Department of Aeronautical Engineering, Chaoyang University of Technology, Taichung 41349, Taiwan; 2 Department of Industrial Engineering and Management, National Chiao Tung University, 1001, University Road, Hsinchu 30010, Taiwan; 3 Department of Computer-Aided Industrial Design, Overseas Chinese University, Taichung 41349, Taiwan

**Keywords:** 3D printing, ubiquitous service, manufacturing lead time, early termination, dental

## Abstract

Three-dimensional (3D) printing has great potential for establishing a ubiquitous service in the medical industry. However, the planning, optimization, and control of a ubiquitous 3D printing network have not been sufficiently discussed. Therefore, this study established a collaborative and ubiquitous system for making dental parts using 3D printing. The collaborative and ubiquitous system split an order for the 3D printing facilities to fulfill the order collaboratively and forms a delivery plan to pick up the 3D objects. To optimize the performance of the two tasks, a mixed-integer linear programming (MILP) model and a mixed-integer quadratic programming (MIQP) model are proposed, respectively. In addition, slack information is derived and provided to each 3D printing facility so that it can determine the feasibility of resuming the same 3D printing process locally from the beginning without violating the optimality of the original printing and delivery plan. Further, more slack is gained by considering the chain effect between two successive 3D printing facilities. The effectiveness of the collaborative and ubiquitous system was validated using a regional experiment in Taichung City, Taiwan. Compared with two existing methods, the collaborative and ubiquitous 3D printing network reduced the manufacturing lead time by 45% on average. Furthermore, with the slack information, a 3D printing facility could make an independent decision about the feasibility of resuming the same 3D printing process locally from the beginning.

## 1. Introduction

3D printing is an advanced computer and information technology that can be used to build a biological object layer-by-layer using a bioprinter controlled by a personal computer [1]. 3D printing has been extensively applied in the medical field, including surgical planning, prosthetics, and other purposes [2,3]. A medical image can easily serve as an input to a 3D printing system [4,5]. The low cost and ease of use of bioprinters have resulted in their widespread use [6], even in undeveloped countries and regions [7,8]. In addition, the model files that define bioprintable objects follow common standards (STL or OBJ) and are easy to distribute and access [9]. Both these factors will help to develop a ubiquitous service [7]. However, there are some limitations of 3D printing applications to this industry [2]:

(1) A bioprinter is required and may be expensive: Some bioprinters can be bought for 1000 USD, while others may cost more than 200,000 USD. The price range is wide [10].
(2) It is not easy to operate a bioprinter: Although a bioprinter is basically highly automatic, it still needs a great deal of human intervention, like setting up, updating, periodical maintenance and calibration, cleaning, leveling, adjusting, and early termination [11].
(3) Some raw materials may be expensive: Consumables for 3D printing include photoresist resins, polymeric substances, hydrogels, metals, and others. To meet the high standards for medical applications, the qualified consumables may be expensive.

According to Chen and Tsai [7] a ubiquitous service is an application of ubiquitous computing in the service sector that has a “design anywhere, serve anywhere, and at any time” paradigm. From this definition, the multidisciplinary nature of a ubiquitous service is clear. In particular, to successfully provide a ubiquitous service, various functionalities such as research and development (R and D), production/service, sales, information technology, and logistics must collaborate. Some related literature on this is summarized in Table 1. Clearly, most of the past efforts were focused on the production functionality.

In the traditional service environment, most capacity is used to prepare for the peak periods that are expected to occur in the future, and this anticipation trades the current budget for future capacity. By contrast, in a ubiquitous service environment, additional, short-term capacity is acquired at the expense of logistics and usage costs, which trades the current budget for the current capacity. Clearly, a ubiquitous service eliminates the time gap. However, a ubiquitous service relies on an efficient and ramified logistics network, which implies that a ubiquitous service is feasible only in a limited number of countries and regions. Nevertheless, this problem can be addressed if the required capacity can be easily built up in any place when required, which is exactly the case with 3D printing [24].

Advanced computer and network technologies have been extensively applied to ubiquitous health care [25]. In this study, a collaborative and ubiquitous additive manufacturing network was designed for fabricating dental parts. The collaborative and ubiquitous additive manufacturing network comprises an Internet of Things [26] and provides a location-based dental-part fabrication service [27]. The collaborative and ubiquitous additive manufacturing network receives a customer’s order online via smart phone or other networking devices [28]. Then, the collaborative and ubiquitous additive manufacturing network searches for 3D printing facilities that are available and located near the customer and distributes the ordered quantity for the 3D printing facilities to fulfill the order collaboratively to minimize the manufacturing lead time. After printing, a transportation service visits the 3D printing facilities one-by-one to pick up the printed dental parts and deliver them to the customer. Fabricating a denture in the traditional way takes about two to three weeks, mostly owing to the shortage of available denturists. The proposed methodology gathers the nearby available capacity for fabricating dentures and is suitable for solving this problem. In addition, the existing 3D printing applications are usually based on a single 3D printing facility. When a 3D printing facility is busy, all unprocessed jobs have to wait, which results in delays in delivering the orders to customers. The proposed methodology is also able to solve this problem. Compared with existing ubiquitous services, such as that proposed by Chen and Wang [17], the collaborative and ubiquitous additive manufacturing network is novel because of the consideration of early termination, which is explained as follows: During a 3D printing process, if the first few layers are not satisfactory, a common practice is to terminate the process early [29]. However, this has rarely been considered by the existing ubiquitous services. The collaborative and ubiquitous additive manufacturing network addresses this concern by designing an efficient resuming mechanism that enables a 3D printing process that has been stopped the early stage to be resumed from the beginning in the same facility only if the optimality of the original printing and delivery plan will not be violated. As a result, the time required for re-negotiation and re-planning can be saved, which is an advantage of the proposed methodology over the existing ubiquitous services. The differences between the established system and some similar systems in the recent literature are summarized in Table 2.

The differences between this study and Chen [31] include:

(1) Fuzzy logic was applied in Chen [31] to consider uncertainty, but was not applied in this study. The uncertainty issue was not taken into account in this study.
(2) 3D printing was applied to different fields in the two studies. It was applied to the toy industry in Chen [31], but to the dental industry in this study.
(3) The applications in the two studies are of different natures. In this study, products made by 3D printing are fully customized. By contrast, in Chen [31], 3D printing was applied to duplicate old toys that are no longer made in the factory.
(4) In addition, the chain effect between two successive 3D printing facilities is considered in this study to gain more slack, which was not considered by Chen [31] and is the novelty of this study.

The remainder of this paper is organized as follows. First, Section 2 is dedicated to the review of past work. The operational procedure of the collaborative and ubiquitous additive manufacturing network is described in Section 3. Then, the mixed-integer linear programming (MILP) and mixed-integer quadratic programming (MIQP) models are proposed to distribute the required pieces and plan the delivery route to minimize the manufacturing lead time, respectively. Subsequently, the slack information is derived and provided to each 3D printing facility so that it can determine the feasibility of resuming the same 3D printing process locally from the beginning. A numerical example is provided to illustrate these steps. Furthermore, to assess the effectiveness of the collaborative and ubiquitous additive manufacturing network, a regional experiment was conducted, which is detailed in Section 4. The performance of the collaborative and ubiquitous additive manufacturing network is also compared with those of several existing methods. Finally, the conclusions and some instructive remarks for guiding future investigations are presented in Section 5.

## 2. Review of Past Work

According to Tuomi et al. [32], biomanufacturing (or tissue engineering), preoperative planning, inert implants, orthodontic treatment, postoperative support structures and surgical special instruments were the main medical applications of 3D printing, showing the importance of 3D printing applications to dental operations. Subsequently, Mäkitie et al. [33] classified the medical applications of 3D printing into five categories: preoperative planning, surgical training and teaching; inert implants; surgical instruments and special equipment associated with the operations; postoperative guides, long-term supports and aids; and artificial tissue. The application of 3D printing to fabricating dental parts emerged in the early 2000s [34]. In the beginning, 3D printing was applied to the single-unit low-volume production of dental implants [35], which could be done at a single 3D printing facility and did not need the cooperation of multiple 3D printing facilities. With the increase in the number of materials (including polymers, metals, and ceramics) and scanning technologies (such as intra-oral scanners) supporting the 3D printing of dental parts, the types of applications become diversified. Thus far, the mainstream applications of 3D printing in this field have included the fabrication of drill guides for dental implants, the preparation of physical models for prosthodontics, orthodontics, and surgery, the manufacturing of dental, craniomaxillofacial and orthopedic implants, and the fabrication of copings and frameworks for implant and dental restorations [36]. Yang et al. [37] applied laser beam melting (LBM) to fabricate porous dental implant prototypes with Ti6Al4V alloy. Cresswell-Boyes et al. [38] printed an artificial tooth from X-ray microtomography (XMT) scans. In this way, the accuracy of the artificial tooth was considerably enhanced. In addition, the application to the dental field can be combined with those to other fields to achieve synergy. For example, Nickels [39] applied selective laser melting (SLM) to design a patient-specific, ready-for-implantation titanium mandible that was combined with dental implants to support a mandibular denture.

In addition, there will be an explosive growth in the dental market for 3D printing applications [40]. In order to meet urgent and large-scale needs, it is more effective to establish a collaborative capacity network. Meanwhile, the CAD/CAM technologies used in dentistry are transiting from closed to open access systems, which provides another motive for establishing a collaborative capacity network, or a ubiquitous manufacturing (UM) network, in the dental market. However, although UM has been receiving much attention in the recent years [13,16], UM networks have not been established in the dental field. These motives drive us to establish a collaborative and ubiquitous additive manufacturing network for the collaborative fabrication of dental parts. The collaborative and ubiquitous additive manufacturing network established in this study is obviously a cloud-based cyber-physical system [41] that can be applied to support the implementation of Industry 4.0 [7,42].

It is often questioned whether the quality of a 3D-printed dental part is comparable to its counterpart made in the traditional way [10,42]. To investigate this issue, Tunchel et al. [43] conducted a three-year follow-up clinical study to evaluate the survival (or success) rates of 3D-printed titanium dental implants. After three years of loading, the survival rate was up to 94.5%, which supported the effectiveness of fabricating titanium dental implants using 3D printing for the rehabilitation of single-tooth gaps in both jaws, at least for a period of three years. Akmal et al. [44] embedded a radio frequency identification (RFID) sensor into a dental implant, so that the dental implant could be easily traced and identified. According to the ISO/ASTM 529000:2015(en) standard, four additive manufacturing technologies, material extrusion, binder jetting, vat photopolymerization, and powder bed fusion, were applied in their study to make a dental implant. The biomaterial for making a dental implant depended on the additive manufacturing technology. Quality control is another issue hampering the establishment of a collaborative and ubiquitous additive manufacturing network. All participants of a collaborative and ubiquitous additive manufacturing network should follow the same quality control procedure to ensure that the dental parts made by them meet the same quality standards. The quality of a 3D-printed dental parts can be assessed in terms of accuracy, biocompatibility, and osteogenic capability [37,38].

## 3. The Collaborative and Ubiquitous Additive Manufacturing Network for Fabricating Dental Parts

**Figure 1:**
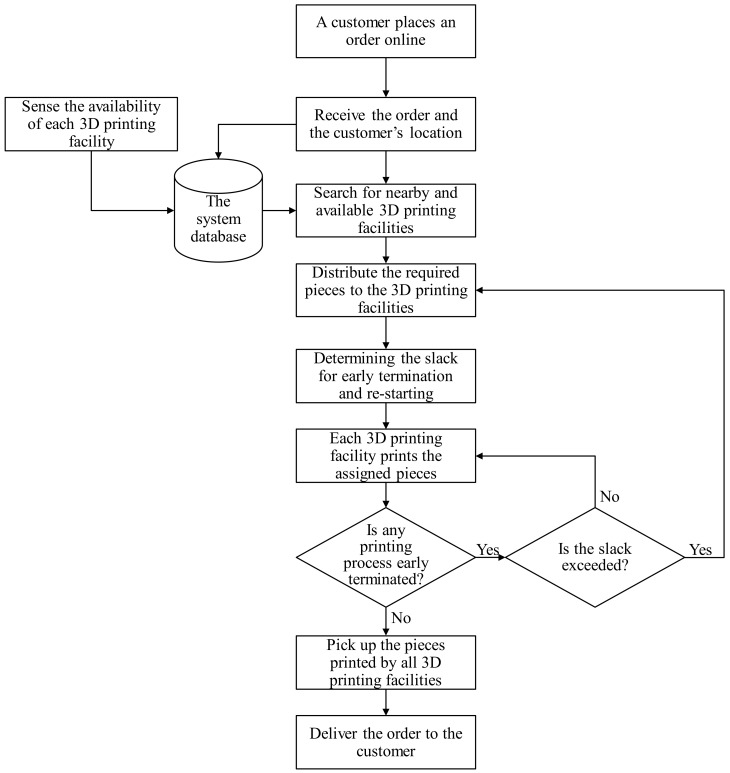
The operational procedure of the collaborative and ubiquitous additive manufacturing network.

The operational procedure of the collaborative and ubiquitous additive manufacturing network for fabricating dental parts is illustrated in Figure 1 and comprises the following steps:

(1) A customer places an order of dental parts online.
(2) The system then searches for 3D printing facilities that are available and near the customer.
(3) The system distributes the ordered quantity for the 3D-printing facilities to fulfill the order collaboratively.
(4) The system determines the slack for early termination and resuming in each 3D printing facility.
(5) Each 3D printing facility prints the assigned pieces.
(6) If any 3D printing process is terminated early, the system progresses to Step 8; otherwise, it progresses to Step 9.
(7) If the slack is exceeded, the system returns to Step 4 to reoptimize; otherwise, it returns to Step 6 to reprint.
(8) A transportation service visits the 3D printing facilities one-by-one to pick up the 3D objects.
(9) The transportation service delivers the order to the customer.

Antunes et al. [45] established a two-tier management platform for Internet of Things applications. The collaborative and ubiquitous additive manufacturing network has a three-tier architecture: mobile user, system (i.e., internet service provider), and 3D-printing facilities.

The variables and parameters used in the proposed methodology are defined as follows:

(1) $a_{i}$: the available time of the *i*-th 3D printing facility; *i* = 1, …, *m*.
(2) $d_{Oi}$: the shortest path length between *O* and 3D printing facility *i*; $d_{Oi}=d_{iO}$.
(3) $d_{ij}$: the shortest path length between 3D printing facilities *i* and *j*; *j* = 1, …, *m*; *j* ≠ *i*. Clearly, $d_{ij}=d_{ji}$.
(4) $n_{i}$: the number of pieces to be printed in the *i*-th 3D printing facility.
(5) $l_{i}$: the time that the transportation service leaves the *i*-th 3D printing facility.
(6) *O*: the start location as well as the destination of the transportation service.
(7) $p_{i}$: the time required to print a piece in the *i*-th 3D printing facility.
(8) $r_{i}$: the arrival time at the *i*-th 3D printing facility.
(9) *t*: the current time.
(10) $X_{ij}$: a state variable. If the transportation service travels from 3D printing facility *i* to 3D printing facility *j*, $X_{ij}=1$; otherwise, $X_{ij}=0$. *j* = 1, …, *m*; *j* ≠ *i*.
(11) $X_{iO}:$ a state variable. If the transportation service returns to *O* from 3D printing facility *i*, $X_{iO}=1$; otherwise, $X_{iO}=0$.
(12) $X_{Oi}:$ a state variable. If the transportation service originates from *O* before travelling to 3D printing facility *i*, $X_{Oi}=1$; otherwise, $X_{Oi}=0$.

### 3.1. Split an Order Multiple 3D Printing Facilities

The decision-making function in the collaborative and ubiquitous additive manufacturing network works by solving mathematical programming problems. Such a treatment has been extensively adopted in the past studies [46].

Only the 3D printing facilities in the proximity of a customer are considered. Balancing the workloads of the 3D printing facilities helps avoid the starvation or congestion of any one 3D printing facility. Therefore, the number of pieces to be printed in each 3D printing facility is determined using the following model:

**(Model I)**

(1) $$\mathrm{Min}\;Z_{1}=\underset{i}{\max}(a_{i}+n_{i}p_{i})$$

Subject to:

(2) $$\sum_{i=1}^{m}n_{i}=N$$

(3) $$n_{i}\in Z^{+}\cup \{0\};\;i\;=\;1,\;\ldots ,\;m$$

The objective function minimizes the maximal completion time, that is, the makespan. Equation (2) requests that the numbers of pieces printed at all 3D printing facilities add up to *N*. The model is an MILP problem.

$Z_{1}$ is the maximum of $a_{i}+n_{i}p_{i}$. Therefore:

(4) $$Z_{1}\geq a_{i}+n_{i}p_{i};\;i\;=\;1,\;\ldots ,\;m$$

The MILP problem becomes:

**(Model Ia)**

(5) $$\mathrm{Min}\;Z_{1}$$

Subject to:

(6) $$Z_{1}\geq a_{i}+n_{i}p_{i};\;i\;=\;1,\;\ldots ,\;m$$

(7) $$\sum_{i=1}^{m}n_{i}=N$$

(8) $$n_{i}\in Z^{+}\cup \{0\};\;i\;=\;1,\;\ldots ,\;m$$

**Figure 2:**
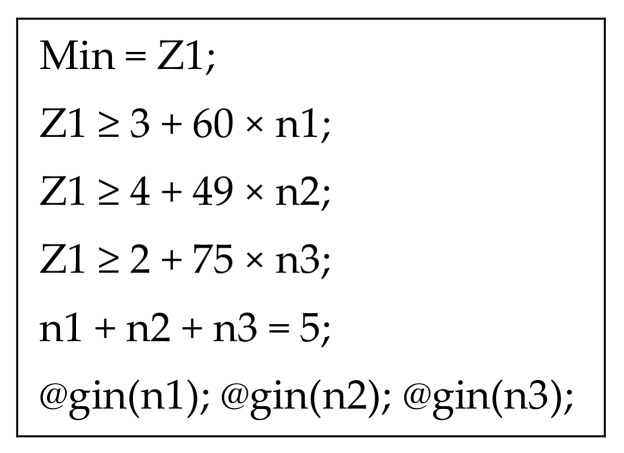
MILP model.

**Example** **1.** *An illustrative example is presented in Table 3. Five pieces of a dental part must be printed by three 3D printing facilities collaboratively. The required MILP model, coded using Lingo, is shown in Figure 2. The optimization result is* $Z_{1}^{*}=123$ *when (* $n_{1}^{*}$ *,* $n_{2}^{*}$ *,* $n_{3}^{*}$ *) = (2, 2, 1).*

### 3.2. Making the Delivery Plan

Subsequently, the pieces printed in different 3D printing facilities must be picked up and then delivered to the customer, which relies on a delivery plan that minimizes the transportation time:

(9) $$\mathrm{Min}\;Z_{2}$$

Subject to:

(10) $$r_{i}\geq X_{Oi}(t+d_{Oi});\;i\;=\;1,\;\ldots ,\;m$$

(11) $$r_{i}\geq X_{ji}(l_{j}+d_{ji});\;i,\;j\;=\;1,\;\ldots ,\;m;\;j\;\neq \;i$$

(12) $$l_{i}\geq a_{i}+n_{i}^{*}p_{i};\;i\;=\;1,\;\ldots ,\;m$$

(13) $$l_{i}\geq r_{i};\;i\;=\;1,\;\ldots ,\;m$$

(14) $$X_{ij}+X_{ji}\leq 1;\;i,\;j\;=\;1,\;\ldots ,\;m;\;j\;\neq \;i$$

(15) $$X_{Oi}+\sum_{j\neq i}X_{ji}=1;\;i\;=\;1,\;\ldots ,\;m$$

(16) $$X_{iO}+\sum_{j\neq i}X_{ij}=1;\;i\;=\;1,\;\ldots ,\;m$$

(17) $$\sum_{i=1}^{m}X_{Oi}=1$$

(18) $$\sum_{i=1}^{m}X_{iO}=1$$

(19) $$Z_{2}=\sum_{i=1}^{m}X_{iO}(l_{i}+d_{iO});\;i\;=\;1,\;\ldots ,\;m$$

(20) $$X_{Oi},\;X_{iO},\;X_{ij}\in \{0,\;1\};\;i,\;j\;=\;1,\;\ldots ,\;m;\;j\;\neq \;i$$

The arrival time at a 3D printing facility is determined by the time at which the transportation service leaves the antecedent facility, which can be *O* or another 3D printing facility, as shown in Equations (10) and (11). The leaving time must be greater than the arrival time and completion time, as required by Equations (12) and (13). Equation (14) ensures the unit-directional property of the transportation plan. Each 3D printing facility can be connected from only a single node, as required by Equation (15), and can be connected to only a single node, as required by Equation (16). Equation (17) ensures that only a single 3D printing facility is visited first, whereas Equation (18) ensures that only a single 3D printing facility is visited last. The manufacturing lead time is determined by the leaving time from the final 3D printing facility visited, as indicated in (19). If the transportation service leaves from 3D printing facility *i* and returns to *O*, then $X_{iO}=1$ and $Z_{2}=l_{i}+d_{iO}$. This model is an MIQP problem. The algorithm proposed in Chen and Wang [17] can be applied to help solve the MIQP problem.

**Figure 3:**
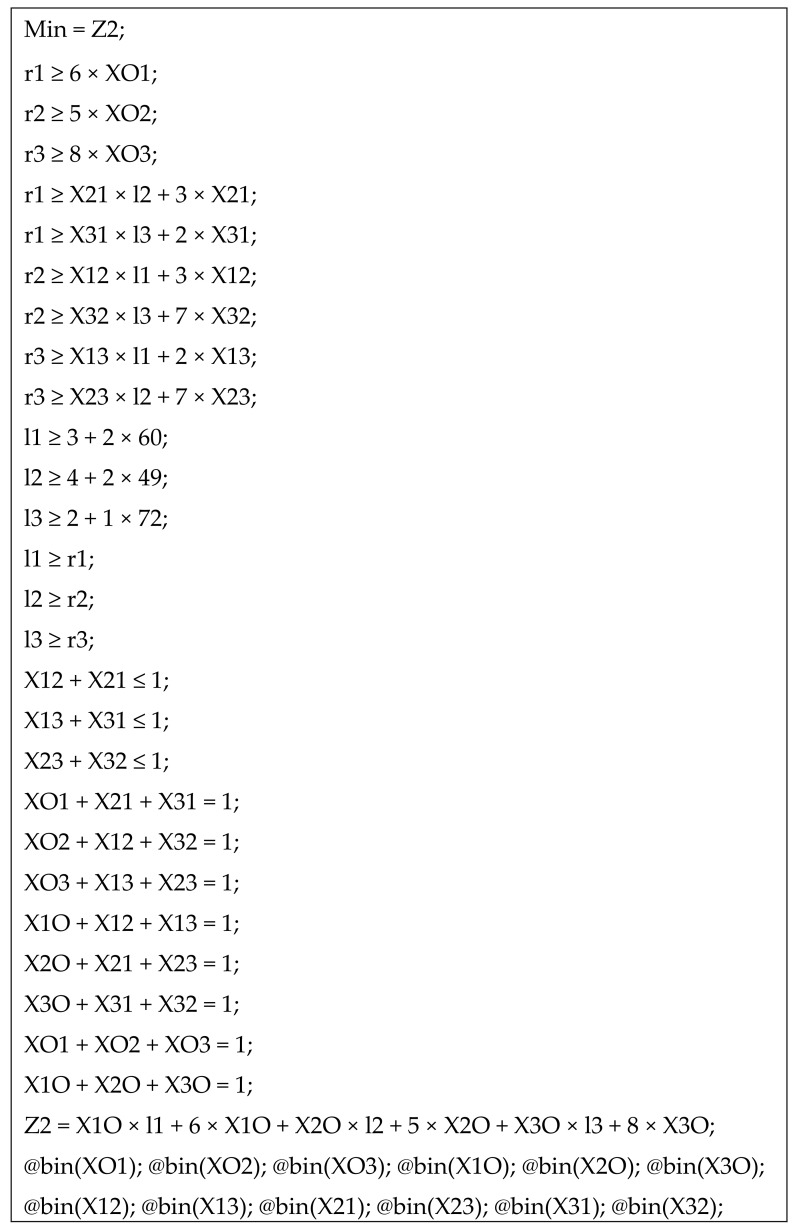
MIQP model.

**Example** **2.** *In the previous example, the distance between every pair of nodes is estimated using Google Maps, and the results are summarized in Table 4, which is called the distance matrix. t = 0. The MIQP model for this example is presented in Figure 3. The optimal objective function value is*$Z_{2}^{*}=129$*when the transportation plan is*O→2→3→1→O.

### 3.3. Determining the Slack for Early Termination and Ressuming by Considering an Individual 3D Printing Facility

It is common for a 3D printing facility to terminate a 3D printing process early if the result is not satisfactory. Early termination should be performed as soon as possible so that the 3D printing process can be resumed from the beginning immediately. However, in a collaborative service setting, after terminating a 3D printing process early, the optimal printing plan may change, which requires a new round of optimization. However, at that time, the conditions of the 3D printing facilities may be completely different. Consequently, a 3D printing process that has been stopped the early stage may have to be resumed from the beginning elsewhere.

To solve this problem, a simple yet effective treatment is to instruct each 3D printing facility the feasibility of resuming the same 3D printing process locally from the beginning. Therefore, a parametric analysis is performed as follows.

When a greater-than or equal constraint is not binding, the surplus is the extra amount over the constraint that is being used. Indicating the surplus of Equation (12) with $S_{i}$ for the *i*-th 3D printing facility:

(21) $$l_{i}^{*}-S_{i}=a_{i}+n_{i}^{*}p_{i};\;i\;=\;1,\;\ldots ,\;m$$

Then, the value of $n_{i}^{*}$ in Equation (21) remains unchanged when $n_{i}^{*}p_{i}$ increases by $S_{i}$. Therefore, if the 3D printing process is terminated early after $\xi_{i}$ min of printing, it can be resumed from the beginning in the same 3D printing facility if:

(22) $$\xi_{i}\leq S_{i}$$

without changing the optimality of the solution. $S_{i}$ is called the slack for early termination and resuming from the beginning in the same 3D printing facility.

**Example** **3.** *The values of the surpluses obtained in the previous example are summarized in Table 5. The results show that in 3D printing facility 3, if the printing process of the dental part is stopped 32 min or earlier after printing begins, it can be resumed from the beginning in the same 3D printing facility. The original solution is still optimal. However, if the printing process fails, for example 33 min after printing began, then it may not be resumed from the beginning in the same 3D printing facility because the original solution may no longer be optimal.*

In project management, float or slack (i.e., the latest start (or finish) time minus the earliest start (or finish) time) is the amount of time that a task can be delayed without delaying the subsequent task or postponing the project completion time [47]. Determining the slack for the early termination and resuming of a 3D printing process is similar to determining the slack of a task in project management. However, there are some differences between them, as summarized in Table 6.

If the entire collaborative and ubiquitous additive manufacturing network is considered, more slack can be gained from the chain effect between two successive 3D printing facilities.

### 3.4. Determining the Slack for Early Termination and Resuming by Considering the Chain Effect

**Figure 4:**
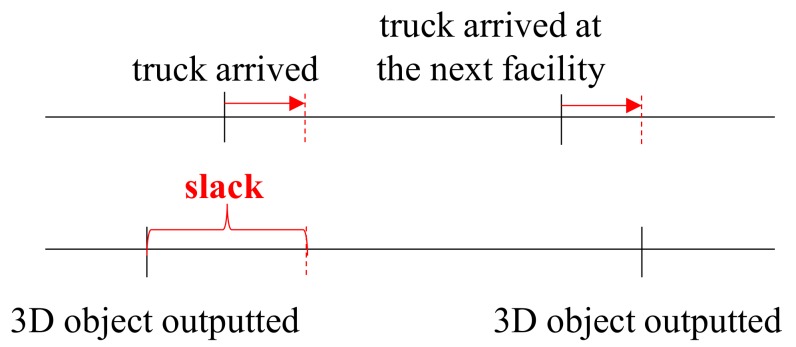
Determining the slack.

More slack can be gained by considering the chain effect between two successive 3D printing facilities. The rationale is that the delivery plan remains optimal if the extended time does not exceed the slack, as illustrated in Figure 4, in which the slack for early termination and resuming from the beginning in the *i*-th 3D printing facility is derived as:

(23) $$S_{\left(i\right)}=\max(r_{\left(i\right)}^{*}-c_{\left(i\right)}^{*},\;0)+\max(c_{\left(i+1\right)}^{*}-r_{\left(i+1\right)}^{*},\;0)$$

**Example** **4.** *In the previous example, the data required for deriving the slack in each 3D printing facility are summarized in Table 7, based on which the following results are derived:*

$$\begin{array}{ll} S_{\left(1\right)} & =\max(5-102,\;0)+\max(77-109,\;0)\\ & =\max(-97,\;0)+\max(-32,\;0)\\ & =0+0\\ & =0 \end{array}\;\begin{array}{ll} S_{\left(2\right)} & =\max(109-77,\;0)+\max(123-111,\;0)\\ & =\max(32,\;0)+\max(12,\;0)\\ & =32+12\\ & =44 \end{array}\;\begin{array}{ll} S_{\left(3\right)} & =\max(111-123,\;0)\\ & =\max(-12,\;0)\\ & =0 \end{array}$$

which implies that in 3D printing facility 3 (that is, the second visited), if the printing process of the dental part is stopped 44 min or earlier after printing begins, it can be resumed from the beginning in the same 3D printing facility without changing the optimal delivery plan.

On the basis of the provided slack information, a 3D printing facility can make a quick and independent decision.

**Theorem** **1.** *The slack calculated using Equation* (23) *is greater than that calculated using Equation* (21).

**Proof.**  If $r_{i}^{*}<c_{i}^{*}$, then $l_{i}^{*}=c_{i}^{*}$. The slack calculated using Equation (21) becomes:

(24) $$\begin{array}{ll} S_{i} & =l_{i}^{*}-a_{i}-n_{i}^{*}p_{i}\\ & =l_{i}^{*}-c_{i}^{*}\\ & =0 \end{array}$$

Whereas that calculated using Equation (23) becomes:

(25) $$\begin{array}{ll} S_{\left(i\right)} & =\max(r_{\left(i\right)}^{*}-c_{\left(i\right)}^{*},\;0)+\max(c_{\left(i+1\right)}^{*}-r_{\left(i+1\right)}^{*},\;0)\\ & =0+\max(c_{\left(i+1\right)}^{*}-r_{\left(i+1\right)}^{*},\;0)\\ & \geq 0 \end{array}$$

Clearly, the slack calculated using Equation (23) is greater than that calculated using Equation (21). By contrast, if $r_{i}^{*}\geq c_{i}^{*}$, then $l_{i}^{*}=r_{i}^{*}$. The slack calculated using Equation (21) becomes:

(26) $$\begin{array}{ll} S_{i} & =l_{i}^{*}-c_{i}^{*}\\ & =r_{i}^{*}-c_{i}^{*} \end{array}$$

Whereas that calculated using Equation (23) becomes:

(27) $$\begin{array}{ll} S_{\left(i\right)} & =\max(r_{\left(i\right)}^{*}-c_{\left(i\right)}^{*},\;0)+\max(c_{\left(i+1\right)}^{*}-r_{\left(i+1\right)}^{*},\;0)\\ & =r_{\left(i\right)}^{*}-c_{\left(i\right)}^{*}+\max(c_{\left(i+1\right)}^{*}-r_{\left(i+1\right)}^{*},\;0)\\ & \geq r_{\left(i\right)}^{*}-c_{\left(i\right)}^{*} \end{array}$$

The slack calculated using Equation (23) is again greater than that calculated using Equation (21). Theorem 1 is thus proven.

## 4. Experiment

**Figure 5:**
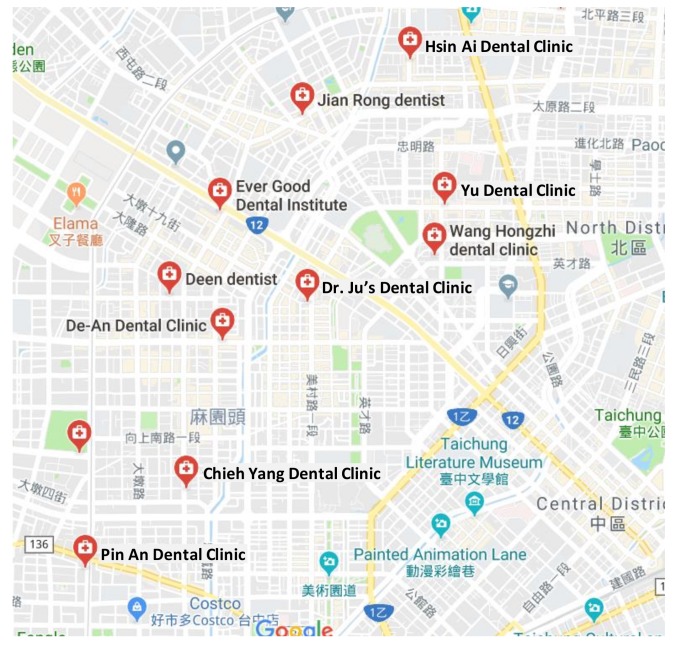
The experimental region.

To the best of our knowledge, the collaborative and ubiquitous additive manufacturing network established in this study is the first attempt in the dental industry. There are no similar cases (with early termination and restarting data) that can be used as benchmarks. For this reason, the effectiveness of the collaborative and ubiquitous additive manufacturing network was assessed through a regional experiment conducted in Taichung City, Taiwan (see Figure 5). The experimental region had an area of approximately 17.8 km^2^ in which there were up to eleven dental clinics. For serving the dental clinics, there were six 3D printing facilities (indicated with *A*–*F*) providing 3D printing networks. In the experiment, each dental clinic placed an order using a Web-based interface. The order was transmitted to the system server. After receiving this, the system server searched the system database to find out nearby 3D printing facilities to print the order in a collaborative way. All 3D printing facilities were available at the beginning of the experiment, however, only the 3D printing facilities within a distance of approximately 20 min to a customer were considered. Therefore, not all of the 3D printing facilities were able to serve each customer. In addition, this study investigated the operations on the system server, including order splitting, production planning, scheduling and re-scheduling, and transportation planning. For these purposes, the participating 3D printing facilities provided various time-related information. The technical details of the participating 3D printing facilities, including the additive manufacturing technologies, 3D printers, and pre-processing and post-processing methods, were not concerned. In fact, the 3D printing facilities for printing different orders might not be the same. A 3D printing facility might even use different 3D printers to print different orders. Nevertheless, the time-related information provided by the 3D printing facilities was the outcomes of their actions.

Ten customers were involved in the experiment. Each customer placed an order of several pieces of a dental part. The dental parts printed in the experiment were mostly denture bases and teeth. The content of the order was transmitted to the system server. On the basis of this information, the system server searched for 3D printing facilities within the proximity of the customer to print the required dental part. The overhead for each order (including confirmation of the availability of each 3D printing facility, completion of the transaction with each 3D printing facility, and decision-making) was restricted to be completed within 20 min. The objective was to minimize the average manufacturing lead time for delivering all orders.

Considering the first customer as an example, the details of his order are summarized in Table 8. There were six 3D printing facilities, named *A*–*F*, near this customer. The time required to print one piece of the ordered dental part in each 3D printing facility is given in Table 9. The distance matrix is shown in Table 10.

The optimization result was {$n_{i}^{*}$} = {0, 0, 0, 0, 1, 1}. The optimal delivery plan was *O* → *F* → *E* → *O*, giving $Z_{2}^{*}=142$ (min). The slacks were derived as $\left\{S_{5},\;S_{6}\}=\{7,\;0\right\}$, indicating that a 3D printing process that terminated early in 3D printing facility E within 7 min could be resumed from the beginning in the same place without changing the optimality of the original printing and delivery plan.

The second customer’s order was distributed for the 3D printing facilities to fulfill the order collaboratively *A*, *B*, *C*, *D*, and *E*, and the dental parts were picked up in the sequence *O* → *D* → *B* → *A* → *C* → *E* → O, resulting in a manufacturing lead time of 223 min. The slacks $S_{1}-S_{5}$ were derived as 31, 18, 80, 0, and 70.8 min, respectively. The 3D printing process in 3D printing facility *C* was terminated 34 min early after printing, which was less than the slack, so the 3D printing process could be resumed from the beginning in the same facility. The manufacturing lead time remained unchanged. Without the slack information, the MIQP model would need to be reoptimized, which would require additional overhead and might lengthen the manufacturing lead time by 20 min.

The third customer placed an order of three pieces that were assigned to 3D printing facilities *D*, *E* and *F*. The pickup sequence was *O* → *F* → *E* → *D* → *O*, and the manufacturing lead time was 214.5 min. The slacks for the three 3D printing facilities were derived as {$S_{4}$, $S_{5}$, $S_{6}$} = {8.9, 0.0, 74.8}. The 3D printing process in 3D printing facility *D* was terminated early at time 14:58 on 2017/8/3, which was 13.2 min after printing began. This was greater than the slack and, thus, the MIQP model was reoptimized. The new printing plan was {$n_{i}^{*}$} = {0, 0, 1, 0, 1, 1}, and the new delivery plan was *O* → *F* → *E* → *C* → *O*. The manufacturing lead time became 246.5 min. By contrast, if the early-terminated 3D printing process had been resumed from the beginning in the same facility, the manufacturing lead time would have been 282.8 min, much longer than the optimal value.

The two pieces ordered by the fourth customer were printed collaboratively by 3D printing facilities *D* and *F*. The delivery plan was *O* → *F* → *D* → *O*, resulting in a manufacturing lead time of 238.4 min. The slacks were 0.0 and 90.8 min, respectively. Both pieces were safely printed without early termination.

The fifth customer ordered three pieces of a dental part at 17:49 on 2017/8/3. 3D printing facilities *C*, *E*, and *F* printed the required pieces collaboratively. Then, the transportation service collected the printed pieces by visiting *E*, *F*, and then *C*. The manufacturing lead time was 165 min. The slacks for the three 3D printing facilities were 18, 0, and 6.5 min. The 3D printing processes of all pieces were successfully completed.

The sixth customer placed his order at 19:16. For him, the optimal production and transportation plan was *O* → *A* → *B* → *D* → *O*. The slacks for the three chosen 3D printing facilities were 0, 13, and 31 min. After successfully completing the 3D printing process, the manufacturing lead time was 85.8 min.

The seventh customer was just 5.7 min behind the six customer. Her order was distributed for the 3D printing facilities to fulfill the order collaboratively C, E, and F, giving a manufacturing lead time of 144 min. The three 3D printing facilities had slacks of 0, 21, and 28 min, respectively. All pieces were successfully printed.

The eighth customer ordered two pieces of a dental part that were printed by 3D printing facilities C and E. The slacks for the two 3D printing facilities were 0 and 20 min, respectively. The two pieces were successfully printed 111 min after placing the order.

At 21:41, the ninth customer placed his order. The optimized production and transportation plan for fulfilling this order was *O* → *A* → *B* → *D* → *O*. After evaluation, the slacks for 3D printing facilities A and B were 0 and 13 min, respectively. In contrast, the slack for 3D printing facility D was up to 90 min. The 3D printing process in 3D printing facility B failed 6.7 min after the process started, which was within the slack. Therefore, the 3D printing process was resumed from the beginning in the same 3D printing facility.

Subsequently, the last customer placed her order that was distributed for the 3D printing facilities to fulfill the order collaboratively C, E, and F. The slacks for these 3D printing facilities ranged from 0 to 81 min. All pieces were successfully printed. The manufacturing lead time for completing the order was 207 min, which was long because of the accumulation of the waiting time.

**Figure 6:**
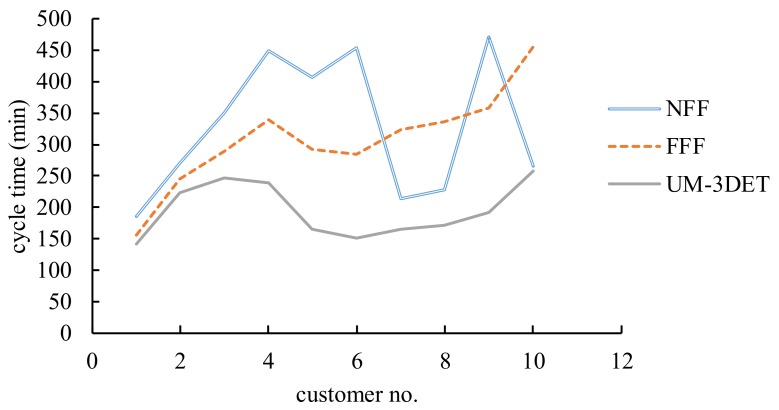
Manufacturing lead times achieved using various methods.

Two existing methods, the nearest-facility-first (NFF) method (i.e., the Google Maps method) and the fastest-facility-first (FFF) method, were also applied to the data of the customers for comparison (Table 11). Clearly, the dispatch, delivery, and restart policies of the two methods are different from those of the proposed methodology. The printing and delivery plans made using the two existing methods are summarized in Table 12. The manufacturing lead times for delivering the orders using various methods are compared in Figure 6.

From the experimental results, the following results were obtained:

(1) Between the two existing methods, the FFF method surpassed the NFF method most of the time, which was not unexpected because printing time is usually longer than transportation time. Therefore, travelling first to a nearer 3D printing facility did not confer much advantage.
(2) Clearly, the collaborative and ubiquitous additive manufacturing network effectively reduced the manufacturing lead times. The advantages over the FFF and NFF methods with respect to the average manufacturing lead time were 35% and 34%, respectively.
(3) In addition, the advantages accumulated over time. For example, compared with the NFF method, the collaborative and ubiquitous additive manufacturing network had 23% lower manufacturing lead time for the first customer, which increased up to 59% for the fifth customer.
(4) To ascertain whether such advantages were significant, paired *t* tests were performed. The results are summarized in Table 13, showing that the manufacturing lead times achieved using the collaborative and ubiquitous additive manufacturing network were statistically shorter than those achieved using the two existing methods at *α* = 0.05.

## 5. Conclusions

3D printing is resulting in evolutionary changes to services. However, studies in this field have been primarily focused on rapid prototyping, that is, the product design phase. Very few studies have been conducted on the ubiquitous service phase. In addition, early termination of a 3D printing process that is destined to fail and then resuming it from the beginning is common practice. Early termination and resuming becomes a critical problem if a 3D printer is used for providing a ubiquitous service. To address this problem, this study established the collaborative and ubiquitous additive manufacturing network. The collaborative and ubiquitous additive manufacturing network is a composite client–server system that distributes the required pieces of an order for several 3D printing facilities to fulfill the order collaboratively. Different from traditional service networks, such as supply chains, only the 3D printing facilities in the proximity of a customer are considered. That is, the participating 3D printing facilities vary from customer to customer and are unknown in advance, resembling the characteristics of a cloud service system.

To fulfill the tasks of dispatch and delivery in the collaborative and ubiquitous additive manufacturing network, a MILP model and a MIQP model were proposed and optimized, respectively. In addition, a slack was derived to determine the longest time for a 3D printing facility to early terminate and resume a 3D printing process from the beginning without violating the optimality of the original printing and delivery plan. The applicability of the proposed methodology was illustrated using a numerical example. Furthermore, a regional study was conducted to validate the effectiveness of the collaborative and ubiquitous additive manufacturing network. According to the experimental results, the proposed methodology exhibited the following advantages over the existing methods:

(1) Compared with the two existing methods, the collaborative and ubiquitous additive manufacturing network reduced the average manufacturing lead time by 35% on average.
(2) The advantage of the collaborative and ubiquitous additive manufacturing network over the two existing methods accumulated over time.
(3) With the slack information, a 3D printing facility can make an independent decision about the feasibility of resuming the same 3D printing process locally from the beginning. In this way, the proposed methodology enabled the distributed and localized decision-making.

However, the proposed methodology also has shortcomings:

(1) It is assumed that the participating 3D printing facilities can achieve the same quality level. Otherwise, the overall quality of the printed pieces cannot be guaranteed, which will discourage customers from using the collaborative and ubiquitous additive manufacturing network.
(2) The pricing policies chosen by different 3D printing facilities are not the same, which needs to be tackled before inviting the 3D printing facilities to join.

Some possible directions for future research are provided as follows:

(1) In this study, the experiment was conducted in a large city. In a smaller city, there are fewer 3D printing facilities, which reduces the size of the MIQP model and increases the efficiency of the system server. However, the manufacturing lead time for delivering an order may be lengthened owing to the limited choices of 3D printing facilities. For this reason, more experimentation must be performed in the future to elaborate the effectiveness of the collaborative and ubiquitous additive manufacturing network.
(2) In addition, if the 3D object to be printed is more complex, the 3D printing process is more likely to fail [48]. The required decision-making also becomes more complicated. Nevertheless, by providing more slack to each 3D printing facility, the proposed methodology is more robust than the existing methods without slack or Chen’s method with limited slack [31].
(3) Further, the proposed methodology can be applied to establish collaborative and ubiquitous systems for other types of products [49]. Although different collaborative and ubiquitous systems adopt different materials and print different products, there is still some overlap between these systems, leaving some space for their cooperation.
(4) Furthermore, the decision-making models proposed in this study can be expanded to incorporate cost- or due-date-related information so that multiple objective functions (such as the manufacturing lead time, total cost, and number of tardy orders) can be optimized simultaneously. Further, the system can be deployed in a cloud-based environment [49,50,51].

## Figures and Tables

**Table 1:** Studies on the various functionalities that assist ubiquitous services.

Ubiquitous Manufacturing Functionality	Related Literature
R and D	Zheng et al. [12]
Production/Service	Lin and Chen [13], Luo et al. [14], Wang, Xie, Zhao, Zhang, and Duan [15], Wang, Wang, Mohammed, and Givehchi [16], Chen and Wang [17]
Sales	Tseng and Hu [18]
IT	Chen and Chiu [19], Cheng et al. [20], Stergiou and Psannis [21]
Logistics	Chen and Lin [22], Luo et al. [14], Nielsen et al. [23], Chen and Wang [17]

**Table 2:** The differences between the established system and some similar systems in the recent literature.

Method	Production Planning	Transportation Planning	Simultaneous Planning	Optimization Method	Slack Consideration	Allowing Early Termination
Chen and Lin [30]	Yes	Yes	No	Heuristic	No	No
Chen and Wang [17]	Yes	Yes	Yes	Branch-and-bound algorithm	No	No
The established system	Yes	Yes	No	Branch-and-bound algorithm	Yes	Yes

**Table 3:** Illustrative example.

*i*	$a_{i}$	$p_{i}$
1	3	60
2	4	49
3	2	75

**Table 4:** Distance matrix.

	*j*	*O*	1	2	3
*i*					
*O*		0	6	5	8
1		6	0	3	2
2		5	3	0	7
3		8	2	7	0

**Table 5:** Surplus values.

*i*	$S_{i}$
1	0
2	0
3	32

**Table 6:** Differences between the proposed methodology and project management.

	Function of Slack	Sequence of Executing Tasks
The Proposed Methodology	For planning the start time	According to the sequence of visiting them
Project Management	For planning the re-start time	No restriction

**Table 7:** Required data for deriving the slacks.

*i*	Facility	$r_{\left(i\right)}$	$c_{\left(i\right)}^{*}$
1	2	5	102
2	3	109	77
3	1	111	123

**Table 8:** The details of the first order.

Customer No.	Detected Location (Latitude, Longitude)	Time	Quantity
1	(24.25, 120.74)	2017/8/3 11:37	2

**Table 9:** Time required to print one piece in each 3D printing facility.

3D printing Facility	Unit Printing Time (min)
*A*	140
*B*	140
*C*	105
*D*	140
*E*	105
*F*	105

**Table 10:** Distance matrix (unit: min).

	*j*	*O*	*A*	*B*	*C*	*D*	*E*	*F*
*i*								
*O*		0	29	27	37	16	30	32
*A*		29	0	13	26	18	13	16
*B*		27	13	0	29	18	16	19
*C*		37	26	29	0	34	20	11
*D*		16	18	18	34	0	23	20
*E*		30	13	16	20	23	0	7
*F*		32	16	19	11	20	7	0

**Table 11:** Policies of various methods.

Method	Dispatching Policy	Delivery Policy	Restarting Policy
NFF	Assign the next piece to the nearest 3D printing facility that is available	According to the dispatching sequence	Restart in the same place
FFF	Assign the next piece to the fastest 3D printing facility that is available	According to the dispatching sequence	Restart in the same place
The collaborative and ubiquitous additive manufacturing network	MILP	MIQP	Restart in the same place if the slack is not exceeded; re-optimize if otherwise

**Table 12:** Printing and delivery plans prepared using the two existing methods.

Customer	NFF	FFF
1	*O* → *D* → *B* → *O*	*O* → *C* → *E* → *O*
2	*O* → *D* → *B* → *A* → *E* → *F* → *O*	*O* → *C* → *E* → *F* → *A* → *B* → *O*
3	*O* → *D* → *A* → *B* → *O*	*O* → *C* → *E* → *F* → *O*
4	*O* → *D* → *B* → *O*	*O* → *C* → *E* → *O*
5	*O* → *A* → *C* → *B* → *O*	*O* → *C* → *E* → *F* → *O*
6	*O* → *B* → *E* → *F* → *O*	*O* → *C* → *E* → *F* → *O*
7	*O* → *A* → *B* → *E* → *O*	*O* → *C* → *E* → *F* → *O*
8	*O* → *A* → *B* → *O*	*O* → *C* → *E* → *O*
9	*O* → *B* → *E* → *F* → *O*	*O* → *C* → *E* → *F* → *O*
10	*O* → *A* → *B* → *E* → *O*	*O* → *C* → *E* → *F* → *O*

**Table 13:** Results of paired *t* tests.

	NFF	FFF	3DUM-ET
Mean	329.54	307.91	195.09
Variation	12,104.52	6085.21	1825.08
Observations	10	10	10
Pearson correlation coefficient	0.139	0.542	
Degree of freedom	9	9	
*t* statistics	3.784	5.442	
P(T ≤ *t*) one-tail	0.002	0.000	
*t* critical one-tail	1.833	1.833	
P(T ≤ *t*) two-tail	0.004	0.000	
*t* critical two-tail	2.262	2.262	
